# First Serologic Analysis of Antibodies Against African Swine Fever Virus Detected in Domestic Pig Farms in South Korea from 2019 to 2024

**DOI:** 10.3390/pathogens14060581

**Published:** 2025-06-11

**Authors:** Seong-Keun Hong, Mugyeom Moon, Ki-Hyun Cho, Hae-Eun Kang, Jong-Soo Lee, Yeon-Hee Kim

**Affiliations:** 1 Foreign Animal Disease Division, Animal and Plant Quarantine Agency, Gimcheon 39660, Republic of Korea; hongsky@korea.kr (S.-K.H.); vet10@korea.kr (K.-H.C.); kanghe@korea.kr (H.-E.K.); 2 College of Veterinary Medicine, Chungnam National University, Daejeon 34134, Republic of Korea; 3 Emergency Centre for Transboundary Animal Diseases, Food and Agriculture Organization of the United Nations, Regional Office for Asia and the Pacific, Bangkok 10600, Thailand; mugyeom.moon@fao.org

**Keywords:** African swine fever virus, antibodies, spatial clustering analysis, ELISA, IPT, South Korea

## Abstract

Background: African swine fever (ASF) is a crucial socioeconomic setback to South Korea’s swine industry. This study aimed to determine seropositivity for ASF virus (ASFV) in pigs that appeared to be infected on farms with reported ASF outbreaks. Methods: A total of 2232 sera from ASF outbreaks (2019–2024) in South Korea were collected. Two enzyme-linked immunosorbent assay (ELISA) kits were used to detect ASFV antibodies, and an immunoperoxidase test (IPT) was used as a confirmatory test following the method recommended by the World Organisation for Animal Health in the Manual of Diagnostic Tests and Vaccines for Terrestrial Animals. Also, spatial clustering was identified using the Density-Based Spatial Clustering of Applications with Noise (DBSCAN) model to understand ASF hotspots in the wild boar population and assess the spatial relationship between the hotspots and ASF antibody-positive domestic pig farms. Results: Antibodies were first detected in Hwacheon in 2020, but by 2024, only 1.43% of pigs had detectable antibodies against ASFV. Although this percentage is still low, the number of antibody-positive pigs is gradually increasing. Additionally, 32 positive samples were found from nine pig farms with outbreaks, and these samples were confirmed positive in both the two ELISA tests and the IPT. The highest seropositivity was recorded at the finishing stage of pig production. When compared to the confirmatory IPT, both blocking and competition ELISA demonstrated high diagnostic sensitivities. The statistical association between ASF antibody-positive farms and wild boars were analyzed using Fisher’s exact test, yielding a significant *p*-value of 0.007. This indicates a strong correlation, as eight out of nine ASF-seropositive farms were located within hotspots that were significantly associated. Conclusions: Our findings provide valuable insights into ASFV antibody detection in South Korea and demonstrate a statistical association between farms housing pigs with ASFV antibodies and hotspots of ASFV-infected wild boars. Confirmatory tests, such as the IPT, are needed. These insights will contribute to the improvement of surveillance and biosecurity measures for swine farms.

## 1. Introduction

African swine fever virus (ASFV) is the etiological agent of African swine fever (ASF), a highly lethal and contagious disease that affects swine populations. Infection in domestic pigs results in significant food insecurity and economic losses for farmers, primarily due to the high mortality rates typically associated with outbreaks. ASFV, which belongs to the genus *Asfivirus* in the family *Asfaviridae*, is categorized as a linear double-stranded DNA virus with a genomic size ranging from 170 to 193 kb and encoding 151 to 167 open reading frames [1,2,3]. The first outbreak of ASF was reported in Kenya in 1921 [4], and the p72 genotype II was first detected in Georgia in 2007. After spreading through Eastern Europe and Russia [5], the disease was first reported in China in 2018 [6], and subsequently spread to neighboring countries in the Asian continent (World Organisation for Animal Health; WOAH WAHIS Interface).

In South Korea, the first ASF notification was reported on 16 September 2019, in the northwestern region of Paju City, Gyeonggi Province. This initial incursion had limited implications in terms of local spread [7]. However, annual reports of ongoing outbreaks have been documented, with an average of 9.8 outbreaks per year. Five years after the onset of the epidemic, more than 4256 cases had been recorded by December 2024 (https://wadis.go.kr (accessed on 2 January 2025)), comprising 49 cases from domestic pig farms (https://www.qia.go.kr (accessed on 2 January 2025)) and the remainder from wild boars. Currently, ASF is considered endemic to wild boar populations in South Korea, leading to sporadic spillovers into pig farms. Despite concentrated governmental efforts to control the movement of wild boars, regions inhabited by infected animals, including confirmed ASF-positive cases, from Paju to Busan have continued to expand eastward and southward [8,9].

The diagnostic evaluation for ASFV can be quite intricate, requiring that the diagnostic assays used are appropriately aligned with the intended purpose of testing. In the acute phase, ASFV genetic material is expected to be detected in blood and tissue samples. The standards for validation are defined by the WOAH [10]. In addition, there are multiple established real-time polymerase chain reaction (qPCR) methods for surveillance as well as individual and herd testing, both commercial kits and published methodologies [11]. The application of molecular techniques for detection may be challenging and necessitates serological testing for animals that have undergone the acute phase, those in the chronic phase, or those infected with attenuated viruses [12,13,14]. Antibodies against ASFV can be detected in pigs that have recovered from infection, typically within 7 to 28 days post-infection; however, antibody formation is often limited during the acute phase of ASFV infection [10]. Based on extensive longitudinal studies of p72 genotype I ASFV infections in Africa and Europe, it is evident that antibodies against ASFV remain detectable for extended durations post-infection [14].

Given the lack of an ASFV vaccine, these antibodies serve as reliable markers of infection status. However, studies in Eastern Africa have reported a diminished prevalence of detectable serological responses to ASFV infection when using the WOAH-approved assays in ASFV-infected pigs [15]. Consequently, the identification of seropositive animals is the most critical and economically feasible strategy for disease management. Serological analysis is the predominant diagnostic technique owing to its simplicity and relatively low cost. In the context of ASF, as vaccines are not yet available, the detection of antibodies is a direct indicator of infection; thus, the identification of these antibodies has significant diagnostic relevance [16,17]. A crucial aspect of ASFV infection is the absence of identifiable serotypes, which may be due to the virus’s inability to generate neutralizing antibodies [18]. This, along with the sustained presence of ASFV-specific antibodies, underscores the critical role of serological approaches in disease management in affected countries.

Most enzyme-linked immunosorbent assay (ELISA) kits target p30 and p72 proteins, although multiple testing options are available [15,19], along with the WOAH recommendations for the implementation of the “in-house” method. The current WOAH-endorsed serological assays for ASF necessitate an initial serum screening conducted via ELISA, followed by either an immunoblotting assay or an immunoperoxidase test (IPT) as a validation measure for any indeterminate ELISA results [11,15,20]. Furthermore, the WOAH recommends that positive or doubtful results obtained from ELISA should be corroborated using the IPT assay, especially when there are concerns regarding the quality of the samples [10]. Previous studies on the outbreak of ASF in South Korea have developed various epidemiological analyses, including geospatial analysis based on topographical characteristics [21]. Additionally, spatial clustering analysis to understand the spread of ASF in wild boars, estimation of the basic reproduction number (R0) through direct and indirect transmission, and the generalized linear logistic regression model have been used to identify environmental factors influencing the formation of some clusters [22]. However, these studies primarily focused on polymerase chain reaction (PCR)-based antigen test results for wild boars and a few infected pig farms, and none have analyzed cases based on the presence or absence of antibodies.

Therefore, this study aimed to test for antibody positivity using sera from pigs on farms with reported ASF outbreaks, using commercial ELISA with antibodies derived from ASFV-affected pigs, and validate the data using an IPT assay. Additionally, we aimed to provide a comparative analysis of the efficacy of these WOAH-approved serological diagnostic test results in relation to spatial clustering analysis.

## 2. Materials and Methods

### 2.1. Viruses and Cell Cultures

A Korean strain of ASFV/Korea/Pig/Paju1/2019 (derived from the spleen of domestic pigs from the first ASF outbreak in South Korea), a virulent haemabsorbing p72 genotype II ASFV, was used for WOAH-antigen production following the method recommended in the Manual of Diagnostic Tests and Vaccines for Terrestrial Animals [10]. The ASFV isolates were propagated and titrated according to a previous study [23], and the isolates were obtained from the procedure of the European Union Reference Laboratory for ASF (CISA-INIA). A Paju1 strain adapted to African green monkey kidney (Vero) E6 cells (ATCC CRL-1586, American Type Culture Collection, Manassas, VA, USA) was used for the production of fixed indirect immunoperoxidase plates [24]. The Vero E6 cells were grown in Dulbecco’s modified Eagle’s medium (DMEM; Thermo Fisher Scientific, Waltham, MA, USA) supplemented with 10% fetal bovine serum (FBS; Thermo Fisher Scientific) and 1% antibiotic-antimycotic (Thermo Fisher Scientific) at 37 °C with 5% CO_2_.

### 2.2. Sampling

In South Korea, the legal framework for controlling outbreaks of ASF is established through the ‘Act on the Prevention of Contagious Animal Diseases’ and the ‘Enforcement Rule for the Control of Exotic Contagious Animal Disease’. Additionally, the Ministry of Agriculture, Food, and Rural Affairs (MAFRA) developed the ‘Standard Operating Procedure (SOP) on ASF’ in August 2018 as a contingency plan and response strategy, based on the aforementioned enforcement rule. According to the ASF SOP, if pigs (regardless of age) die suddenly without symptoms or show signs such as high fever, lethargy, or cyanosis, all animals on the same farm must undergo a thorough clinical examination. Suspected ASF cases should then be identified, and samples must be collected and sent to a designated diagnostic laboratory for confirmatory testing. A panel of 2232 serum samples collected from outbreak pig farms between 2019 and 2024 was used in this study. The outbreak farms were distributed across several regions: Incheon (1 region), Gyeonggi (5 regions), Gangwon (9 regions), and Gyeongsangbuk (4 regions). The number of serum samples collected from each outbreak farm is presented in Table 1. Sampling was conducted at the biosafety level 3 (BSL-3) animal facilities of the Animal and Plant Quarantine Agency (APQA). Forty-nine domestic pig farms were affected by ASFV outbreaks, of which 15 farms were identified through active surveillance and 34 farms had confirmed ASFV infections based on farmer’s notifications. During active surveillance or in instances where the farm owner’s notification was involved, animals suspected of ASFV infection or exhibiting clinical symptoms on farms with reported outbreaks were selectively sampled, and the samples were submitted either in syringes or serum tubes for antibody testing by provincial veterinary services with jurisdiction. After antigenic confirmation, all samples were promptly transferred to the National Reference Laboratory for ASF at the APQA the day after the outbreak for serological testing.

### 2.3. Antibody Detection Using Serological Testing

#### 2.3.1. ELISA

To detect antibodies against ASFV, two commercially available ELISA kits were used according to the manufacturer’s instructions (Ingezim PPA COMPAC, Prod Ref: 11.PPA.K3 (bELISA; Gold Standard Diagnostics, Madrid, Spain), and ID. Screen ASF Competition (cELISA; Innovative Diagnostics, Grabels, France). Ingezim used a purified protein extract from ASFV (VP72) as the antigen, and ID. Screen was used to detect antibodies against the p32 ASFV recombinant protein. The bELISA kit showed 99% diagnostic sensitivity and 100% diagnostic specificity, in addition to 100% analytical sensitivity for detecting ASF antibodies. Also, the cELISA kit demonstrated 100% sensitivity and specificity. To determine the ELISA results, the absorbance was measured at 450 nm using a Tecan Sunrise spectrophotometer (Tecan, Männedorf, Switzerland) and analyzed using Xfluor 4.5.1 software (Tecan, Männedorf, Switzerland). Afterward, the *Blocking* percentage for the Ingezim ELISA results was calculated as follows:

(1) $$Blocking\;\%\;\left(x\%\right)of\;a\;sample=\frac{\mathrm{NC}-\mathrm{Sample}\;\mathrm{OD}}{\mathrm{NC}-\mathrm{PC}}\times 100$$

Result interpretation: *x*% ≥ 50%, positive; *x*% < 40%, negative; 50% > *x*% ≥ 40%, doubtful. The ID. The screen competition percentage (*S/N*%) was calculated using the following formula:

(2) $$S/N\%\;of\;a\;sample=\frac{Sample\;OD-PC}{NC-PC}\times 100$$

Result interpretation: *S/N*% ≤ 40%, positive; *S/N*% ≥ 50%, negative; 40% < *S/N*% ≤ 50%, doubtful. Two commercial ELISA kits were used to improve ASF antibody detection and confirm positive samples through cross-validation. However, to increase the stringency of this study, samples in the inconclusive range were classified as positive and subjected to confirmatory testing.

#### 2.3.2. IPT Assay

The WOAH recommends the indirect IPT, indirect fluorescent antibody testing, and immunoblotting testing as methods for confirmatory testing in the event of an ELISA-positive result [10,17]. The IPT assay is an EURL-prescribed and validated method and was used for confirmatory testing of sera with positive or inconclusive results following ELISA [25]. The IPT was performed using fixed VERO E6 cells infected with a Korean isolate following the procedure described in COS-1 cells by Gallardo et al. [14,26]. Briefly, a blocking solution of phosphate-buffered saline (0.05% Tween-20, 5% skim milk) (PBST) was added to a 96-well plate coated with ASFV-infected Vero E6 cells and incubated at 37 °C for 1 h. During incubation, serially diluted control (ID.vet, MRI-ASF) and sample sera were added to the wells and incubated at 37 °C for 45 min. Then, the plate was washed three times with PBST, and the HRPO-labeled protein A (PIERCE, Thermo Fisher Scientific) was added after diluting in PBST. The plate was incubated at 37 °C for 45 min. The plate was washed as described above. Then, the AEC (3-amino-9-ethylcarbazole) substrate (ENZO Life Sciences, Farmingdale, CT, USA) was added to the wells and incubated at room temperature for 10 min. A color change was observed in the control wells, and PBS was added to stop the reaction. The wells were observed under an optical light microscope, and red cytoplasmic staining indicated the presence of ASFV-positive complexes.

### 2.4. Spatial Clustering Analysis

For wild boars, this dataset encompassed the date of sample collection, geographical coordinates of the collection sites, and ASFV antigenic test results obtained by qPCR from the National Institute of Wildlife Disease Control and Prevention of South Korea. For this study, the extracted surveillance data comprised 4207 ASF-positive wild boars from 2019 to 2024, with the aim of analyzing the association between wild boars and pig farm outbreaks. We performed spatial clustering analysis to identify hotspots (clusters of ASFV-positive wild boar) using the Density-Based Spatial Clustering of Applications with Noise (DBSCAN) model [27] and Python software (Python Software Foundation, Python Language Reference ver. 3.9.21 retrieved from https://www.python.org/ (accessed on 3 December 2024)). The optimal epsilon (0.6) was determined from the k-distance graph using the elbow method [27]. Through comparative analysis, the minimum number of points required to form a cluster was set to three, which minimized noise and enhanced the precision of the cluster analysis. Based on the results of the initial clustering analysis, a secondary spatial clustering analysis was conducted to identify specific ASF outbreak hotspots in wild boars. Likewise, the DBSCAN method was applied with the same minimum of three points but with a re-optimized epsilon value of 0.25, which fit wild boar movement and ecology patterns [21,28]. The spatial extent of wild boar activity for each identified cluster was estimated as a circular buffer zone, using the centroid of each cluster as the center and the maximum distance from the centroid to any point within the cluster as the radius.

### 2.5. Statistical Analysis

The assay agreement between the ELISA and IPT was evaluated using the hierarchical kappa-type coefficient (κ value). The κ value was calculated using the formula and was interpreted according to the Landis and Koch descriptors [29]. The tests were compared with respect to diagnostic sensitivity, specificity, and the κ coefficient, utilizing the IPT as the reference assay. The results were classified according to the κ value as follows: κ ≤ 0, considered to represent poor; 0 < κ ≤ 0.2, slight; 0.2 < κ ≤ 0.4, fair; 0.4 < κ ≤ 0.6, moderate; 0.6 < κ ≤ 0.8, substantial; and 0.8 < κ ≤ 1, almost perfect. The locations of ASF events on domestic pig farms were overlapped with the circular buffers estimated based on the hotspots of ASF-infected wild boars, the results of DBSCAN analysis. A contingency table was prepared to examine the statistical association between the location of farms with ASF antibody-positive pigs and their inclusion within the circular buffers estimated from ASF-infected wild boars. Fisher’s exact test was performed to assess the statistical significance of the association, and results were considered statistically significant when the *p*-value was less than 0.05 [30].

## 3. Results

### 3.1. Proportion of ASFV-Seropositive Pigs on Pig Farms Determined by ELISA

We evaluated the kinetics of ASFV-specific antibody responses in serum using different commercial ELISA methods with ASFV p72 and p32 antigens coated on the plates. Antibodies were first detected in pigs from an ASF-infected farm in Hwacheon, Gangwon Province, in October 2020. In 2021, no antibodies were detected in the sera of 145 pigs from the five farms. However, in 2022, antibodies were detected in five samples collected from two farms. In 2023, three pigs tested positive on one farm, and in 2024, 23 samples were confirmed to be positive on five farms with reported ASFV outbreaks. With respect to the region, the ASFV seropositivity rate in Gangwon Province was approximately 2% each year; however, it significantly increased to 6.42% (21/327) by 2024. In addition, ASF was first reported in Gyeongsangbuk-do in 2024; however, given that antibodies were detected in two out of 224 samples that tested positive, it is plausible that the antibody detection rate may have increased, similarly to the pattern observed in Gangwon Province. Among these regions, the highest number of antibody-positive results was observed in Hwacheon, Gangwon Province (12/266, 4.51%), followed by Hongcheon (8/209, 3.83%), Cheorwon (5/260, 1.89%), Yangyang (3/30, 10%), Yanggu (2/25, 8%), Andong, Gyeongsangbuk-do (1/18, 5.56%), and Yeongcheon (1/166, 0.6%). In contrast, no cases were detected in Gyeonggi-do (Table 1). Upon comparing the stages of pig production, the samples comprised 28.54% sows, 5.20% gilts, 42.79% finishing (including growing) pigs, 20.47% nursery pigs (with piglets), and 0.31% boars. Of these, 0.78% sows, 2.59% gilts, 2.41% finishing pigs, and 0.22% nursery pigs were positive for ASFV antibodies based on the antibody assay. The finishing (including growing) phase of pig production showed the highest seropositivity, with 23 antibody-positive cases detected (23/955) (Table 2). As for antibody detection, the bELISA detected 37 out of 2232 (1.66%) positive or inconclusive serum samples. Of the 675 screening samples suspected to be positive, 31 (4.59%) tested positive on the cELISA. Three of the screened and confirmed seropositive samples were ASFV qPCR-negative.

### 3.2. Comparison of the ELISA and IPT Results

**Figure 1:**
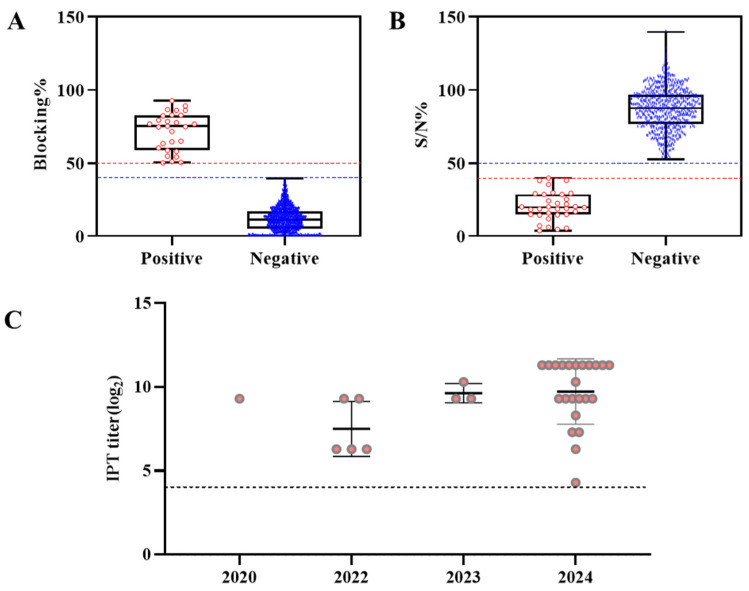
Distribution of ASFV-specific antibodies in pig serum samples collected from ASF outbreak farms in South Korea, classified according to the results from two types of commercial ELISA kits and the WOAH reference for IPT titration. (**A**) Results from the Ingezim ELISA kit (Blocking%, *n* = 2232) and (**B**) result from the ID.vet competition ELISA kit (competition percentage; S/N%, *n* = 675). The red dashed line represents the positive cutoff value, and the blue dashed line represents the negative cutoff value. (**C**) Individual sera that were positive or doubtful in the ELISA assay (*n* = 32, expect for 8 negative) were examined for antibodies against ASFV by the IPT at the yearly time point. Data are represented as the mean ± SD. Sera with IPT titers ≥ 1:20 are considered positive and the dashed line show the limits of detection. There were no seropositive samples in 2019 and 2021.

Serum samples collected from 49 ASFV-infected pig farms in South Korea between September 2019 and December 2024 were tested for antibodies using commercial ELISA kits. For serological analysis, samples from all farms with reported ASFV outbreaks and varying outbreak dates were collected and evaluated using two distinct methodologies: blocking ELISA (*n* = 2232) and competition ELISA (*n* = 675). The comparison results of all samples, including the positive, inconclusive, and negative samples, are presented in Table 3. Finally, all samples were tested in duplicate using the IPT. The distribution of the %blocking ratio categorized by sample classification is illustrated in Figure 1A. Results from the bELISA indicated that 2232 serum samples were analyzed, yielding a κ value of 0.94, which demonstrates a considerable concordance between the bELISA and IPT. The results demonstrated that the bELISA exhibited a diagnostic specificity of 99.86% and a sensitivity of 100%, both of which were high at the established cutoff. In parallel, the results from the cELISA revealed that 675 serum samples showed consistent outcomes between the cELISA and IPT. The %S/N ratios for all categorized samples are shown in Figure 1B. The κ value between the cELISA and IPT was determined to be 0.92, indicating a substantial level of concordance. At the suggested cutoff, the cELISA kit possessed a specificity of 99.38% and a sensitivity of 96.67%.

### 3.3. Spatial Clustering Analysis with Seropositive Pigs on Pig Farms

**Figure 2:**
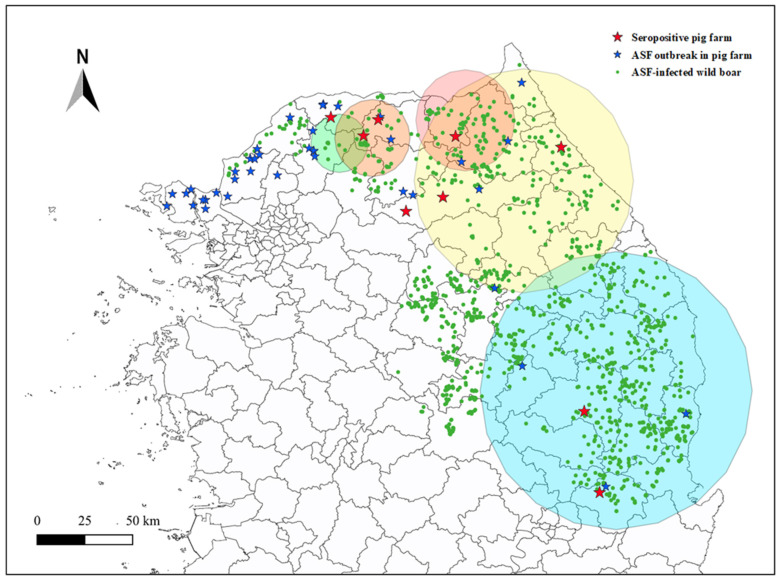
Spatial circular buffer derived from ASFV-infected wild boar clusters and the location of ASF antibody-positives in pig farms: ASF outbreak in pig farms (blue star), seropositive farms (red star) and wild boars (green dot) in South Korea from September 2019 to December 2024. The cluster analysis was conducted, using DBSCAN Model through Python software and the circular buffers were plotted using QGIS Prizren version 3.34.1 (https://qgis.org). The five circular buffers are highlighted with different colors (refer to Table 4 for detailed data).

Five clusters were identified in the first spatial clustering analysis. Most of the 1460 ASF-infected wild boars belonged to a single cluster, representing a hotspot. Subsequent spatial clustering analysis focusing on the main cluster of the initial clustering analysis identified 20 clusters, each varying in the size and density of ASF-infected wild boar points. A circular buffer was generated for each cluster to approximate the spatial extent of wild boar activity, based on the clustering results of ASF-infected wild boar hotspots. Notably, five of these circular buffers contained seropositive domestic pig farms. The number of positive wild boar cases within each cluster ranged from 10 to 608, whereas the cluster radii ranged from 0.165 to 0.787 (Table 4). As shown in Figure 2, eight of the nine ASF-seropositive pig farms were located within these circular buffers, whereas 14 of the 40 antibody-negative farms were included within these clusters. Fisher’s exact test indicated a statistically significant association between the location of ASF antibody-positive farms and their inclusion within the circular buffers (*p* = 0.007). This finding suggests that the presence of ASF antibodies was not independent of the spatial range of wild boar activity zone.

## 4. Discussion

ASFV is a contagious and often fatal disease that affects *Suidae* worldwide. Understanding seropositive dynamics is crucial for an effective response to ASF and requires surveillance of the initial sources of infection, whether on farms or in wild boars, as well as a continuous study of the variations in pathogenesis associated with the dominant genotype viruses on the Korean Peninsula. In Korea, where soft tick vectors are absent, wild boars are known to be hosts that can have a relatively wide range of activity and engage in nocturnal behaviors, allowing them to approach pig farms [31]. The National Institute of Environmental Research officially announced, through a press release on 3 June 2020, that the antibody was first detected in a wild boar captured using a trap in Goseong, Gangwon Province. However, no further information on antibody detection was provided. This is likely because wild boars are generally found as carcasses, and serological testing for ASFV in these animals is not feasible [8,9]. Therefore, further studies are necessary to ascertain the optimal locations for effective and efficient ASFV surveillance activities in districts not included in this analysis, which should include assessments of the utilization of serology in wild boars. This study provides one of the first comprehensive assessments of the association between the presence of ASFV antibodies in pig sera sampled from farms with reported outbreaks across multiple regions, including four provinces and 19 cities and counties in South Korea, and the distribution of ASFV-infected wild boars by analyzing spatial patterns. Additionally, sera from the farms with reported outbreaks were evaluated based on serological significance to confirm the presence of antibodies. In South Korea, ASFV genes are consistently detected every month in wild boars. The seasonal pattern of ASF outbreaks in South Korea, which has four distinct seasons, differs from that observed in other countries [32]. Initially, ASF outbreaks were concentrated in swine farms during the autumn months of September and October, starting in Paju in 2019 and continuing through 2020. However, from 2021 onwards, outbreaks were also detected in May and August. In 2022, outbreaks were reported in November. In 2023, outbreaks occurred primarily between January and April, with an additional outbreak occurring on a farm in July during summer. In 2024, ASF was detected in both June and December, indicating that ASF outbreaks occur year-round throughout the country.

Outbreaks of ASF in pig farms and surveillance data of wild boars show regional differences; specifically, ASF outbreaks in domestic pig farms have been consistently reported in Gyeonggi Province in 2024, whereas ASF antigens in wild boars are increasingly detected only in the southeastern region. This poses limitations for identifying spatial clusters. Some of the identified clusters cover broad areas, whereas others represent dense hotspots. This suggests that wild boar monitoring should be strengthened. Given that ASF has been sporadically and continuously detected in wild boars for over 5 years, irrespective of time and region, spatial cluster analysis can be applied to identify hotspots without considering temporal factors. Therefore, in this study, the statistical analysis of the association between pig farms with detected ASF antibodies and circular buffers of spatial ranges of wild boar activity revealed significant results. This finding suggests that the virus was amplified and circulated extensively in wild boar hotspots, increasing the likelihood of ASF virus exposure on pig farms. The detection of ASF antibodies in pig farms may indicate a potential risk from adjacent wild boar hotspots, as the presence of such hotspots suggests active ASFV circulation in the wild boar population and a higher probability of virus spillovers into nearby farms. Therefore, it is critical to enhance biosecurity and surveillance activities, particularly in areas identified within or near wild boar activity zones and circular buffers. Conversely, close monitoring of the ASFV dynamics in wild boar populations should inform and be integrated into surveillance activities for domestic pig farms. As pointed out, the location of ASFV detection in wild boars and domestic pig farms showed a discrepancy, which posed challenges in our study. This challenge might be caused by limited coordination and communication between ministries. To improve understanding of ASF transmission at the interface of wild boars and domestic pigs and implement more effective control and prevention of ASF, it is essential to establish stronger coordination mechanisms between ministries, facilitate timely data sharing, and regularly update surveillance strategies and plans. These measures will form the foundation for future studies, enabling the identification of more robust clusters and providing deeper insights into the virus transmission dynamics between wild boars and pig farms. This study relied solely on ASF cases, as it was not feasible to obtain comprehensive wild boar population data. In this context, the DBSCAN method was chosen because it does not require the control data and is considered to be suited for identifying clusters based on the spatial distribution of cases alone. However, since all wild boar ASF cases were detected in mountainous areas, the resulting irregular-shaped clusters were confined only within the mountain area. This spatial constraint posed a limitation in directly assessing the relationship between wild boar ASF hotspots and the location of domestic pig farms with ASF antibody detection. As DBSCAN forms clusters based on point density and connectivity, the identified clusters may not accurately reflect the broader spatial extent of wild boar activity. Consequently, areas that potentially fall within the wild boar activity range, such as the outskirts of mountains and nearby agricultural or inhabited areas where pig farms are likely to be located, may not have been fully included in the DBSCAN cluster outputs. To address this, circular buffer zones were generated, using the centroid of each cluster as the center and the maximum distance from the centroid to any point within the cluster as the radius. This approach was used to approximate a more inclusive spatial range of wild boar movement, potentially encompassing both the mountainous and surrounding areas. While this method allowed this study to assess the relationship between the spatial range of wild boars and the location of domestic pig farms with ASF antibody detection, it represents a simplified approximation of wild boar ecology and movement patterns, and should therefore be regarded as a methodological limitation of this study.

In this study, ASFV antibody detection was conducted using commercial ELISA kits from Ingenasa and ID.Vet, which target viral proteins such as p72, p30, and p54. Two different ELISA kits (Ingezim and ID.Vet) were used in parallel to improve diagnostic reliability and to compensate for possible false positives or false negatives associated with a single test. The use of both kits also enabled cross-validation of results and improved confidence in seropositivity detection under field conditions. Positive or inconclusive ELISA results were subsequently validated using the immunoperoxidase test (IPT), the gold standard recommended by the WOAH. Although ELISA demonstrated high sensitivity, inconsistencies—including both false positives and false negatives—were observed, highlighting the need for confirmatory testing for accurate diagnosis. Although the IPT offers high reliability, its reliance on live virus and biosafety level 3 facilities makes it labor-intensive and less feasible in routine field settings. This highlights the pressing need for more accurate and practical serological methods for ASF surveillance.

In the context of farm outbreaks, local livestock control officers are tasked with implementing emergency biosecurity measures. However, due to personnel limitations, the process of selecting suspected animals, collecting blood, and transporting samples for laboratory analysis often becomes a secondary priority. While whole blood is generally collected in EDTA tubes and transported without issue, serum samples are frequently submitted in syringes, reflecting a continued underestimation of their diagnostic importance. This practice has compromised sample quality—such as hemolysis or cross-contamination—and ultimately led to inaccurate serological results. For instance, of the 2232 serum samples analyzed in this study, 10 false positives were attributed to improper sample handling and suboptimal storage conditions.

These findings are consistent with previous studies showing that the quality of field samples significantly impacts diagnostic sensitivity. To address this issue, we have continuously emphasized the importance of using appropriate serum tubes and standardized storage protocols. Notably, proper sample handling practices were widely implemented in 2024, five years after the initial ASF outbreak in South Korea. The improved sample quality from this point onward likely contributed to the observed increase in ASFV seropositivity. As shown in Figure 2, this factor may partially explain the absence of distinct spatial clusters in outbreak areas from 2019 to 2021.

Although the screening assay involved ELISA, which exhibits high sensitivity, prior research has indicated its propensity to yield false negative results [33]. Within the range of commercially available antibody ELISA kits, the predominant testing method is the Ingezim competition ELISA. The manufacturer has reported a sensitivity of 99% and a specificity of 100%, yet other studies have indicated the occurrence of false positives [16]. This discrepancy highlights the importance of further validation and the potential need for supplementary diagnostic techniques to ensure accuracy. In this study, the results included a few false positives. Among the pigs suspected to be positive based on two different ELISA results, five pigs were positive or doubtful based on results from the Ingezim PPA COMPAC kit but tested negative based on results from the IPT, whereas seven pigs were positive based on results from the ID.Vet competition kit. Thus, to confirm suspected positive cases by ELISA, it is essential to conduct a confirmation test using the IPT, which is the gold standard recommended by the WOAH. This test should be conducted at a specialized diagnostic institute such as the National Reference Laboratory or veterinary service with jurisdiction.

Previous investigations have indicated that the insertion, deletion, or mutation of genes in domestic ASFV isolates from swine farms do not affect antibody detection [34]. Among the 49 ASFV isolates obtained from domestic pig farms, almost all were identified as belonging to the p72 (*B646L*) genotype II, Intergenic Region (*I73R* and *I329L*; IGRs) II, except for one isolate from IGR I and one from IGR III, and serogroup 8 (*EP402R*). However, whole-genome analysis revealed no distinctive mutations impacting pathogenicity, even though the virus continues to evolve, albeit at a slow rate [34]. Conversely, based on our previous study, in which we confirmed the pathogenicity of domestic isolates by oral, nasal, and direct transmission in experimental groups, we observed a delayed onset of viremia in animals challenged via the oral route or in sentinel groups compared to those injected intramuscularly. Although none of the animals survived, the time to mortality was delayed in the group that was infected via the oral route and in the sentinel groups [35]. This suggests that direct contact transmission is more likely to induce antibody formation, although antibody testing was conducted in a previous study and the results are not presented. As shown in Table 2, a high antibody-positivity rate of 23 pigs (2.41%) was observed in the fattening production stage. Up until now, the majority of outbreaks in domestic farms have occurred on farrow-to-finish pig farms (39/49, 79.59%). There have also been cases reported on finishing pig farms (6/49, 12.24%). Although the sample size was larger, leading to a higher likelihood of antibody positivity, the transmission potential of the virus was higher in fattening pigs compared to sows, as fattening pigs are not systematically managed in stalls like sows and are housed in groups within pigsties. In these environments, direct contact is more likely, and biosecurity measures are more challenging. As a result, transmission between the pigs may have been easier, and a significant number of deaths occurred prior to the farmer’s notification.

According to our previous studies, qPCR using whole blood allows for rapid and accurate diagnosis and has demonstrated superior sensitivity compared to other sample types [36]. However, confirmatory serological testing is crucial for evaluating swine for ASF exposure. Owing to its severe pathogenicity, most swine afflicted with virulent ASFV succumb prior to developing sufficient protective immunity. In contrast, some seropositive animals from high-mortality ASFV isolates, which correspond to virulent strains, tested PCR-negative, suggesting that they recovered from the infection and that virulent strains may have attenuated or remained subclinically infected [16,37]. Our findings also confirm that in cases where the antigen is negative, and the acute form of the virus infects pigs on the farm, antibodies may still be detected in some pigs. Considering this point, it seems plausible that even acute and highly virulent viruses can subtly infect pigs and induce antibody production.

Given these circumstances, the results of ASF antibody testing are crucial for the implementation of effective biosecurity measures to prevent viral transmission. Our findings suggest that as long as ASFV antigens continue to be detected in wild boars, there is a high likelihood of changes in viral pathogenicity, and the number of antibody-positive pigs will steadily increase. Furthermore, the dominant ASFV strain in neighboring countries shows signs of recombination [38,39,40], and if such recombinant or low-pathogenic strains enter the country, the antibody prevalence can fluctuate. Therefore, continuous antigen-antibody surveillance is essential to prepare for potential changes in ASF dynamics.

## 5. Conclusions

This study presents, for the first time, valuable insight into the spatial relationship between circular buffers of wild boar movement ranges related to hotspots of ASF-infective wild boars and domestic pig farms with serological evidence of ASFV exposure in South Korea. This study observed a statistically significant association between these buffers and the location of ASFV-seropositive pig farms. The findings suggest a statistical correlation between domestic pig farms located near wild boar ASF hotspots and increased antibody positivity, underscoring the potential role of wild boars in ASFV transmission. However, it is important to note that antibody assays using ELISA may yield false positive and false negative results, which should be taken into consideration when interpreting these findings. Consequently, it is imperative to refrain from drawing definitive conclusions regarding antibody positivity exclusively based on ELISA findings. This underscores the necessity for supplementary confirmatory assays, such as the IPT, in addition to ELISA. Collectively, this study provides important information regarding seropositivity on swine farms and effective surveillance to facilitate biosecurity.

## Figures and Tables

**Table 1:** Serological status of domestic pig sera samples collected from ASFV outbreaks in South Korea. ASF-seropositive cases and the regions where ASFV outbreaks occurred from 2019 to 2024.

Province	Region	Collection Date	Num. of Sera	Antibody Detection ^1^				% Sero- Positivity
				Positive	Negative	%SE ^2^	%SP ^3^	
Incheon	Ganghwa	September 2019	36	0	36	-	100	-
Gyeonggi	Gimpo	September 2019 to August 2024	235	0	234	-	99.57 (1)	-
	Yeoncheon	September to October 2019	35	0	35	-	100	-
	Paju	September 2019 to January 2024	212	0	212	-	100	-
	Pocheon	January to April 2023	440	0	440	-	100	-
	Yangju	December 2024	120	0	120	-	100	-
Gangwon	Goseong	August 2021	65	0	65	-	100	-
	Yanggu	August 2022	25	2	23	100	100	8.0
	Yangyang	February 2023	30	3	23	100	82.61 (4)	10.0
	Inje	August to October 2021	40	0	40	-	100	-
	Cheorwon	November 2022 to May 2024	265	5	260	100	100	1.89
	Chuncheon	September 2022	30	0	30	-	100	-
	Hongcheon	August 2021 to November 2024	209	8	198	100	98.48 (3)	3.83
	Hwacheon	October 2020 to October 2024	266	12	253	91.67 (1)	99.60 (1)	4.51
Gyeongsangbuk	Andong	July 2024	18	1	16	100	93.75 (1)	5.56
	Yeongdeok	January 2024	20	0	20	-	100	-
	Yeongcheon	June to August 2024	166	1	165	100	100	0.60
	Yecheon	July 2024	20	0	20	-	100	-
Total			2232	32	2190	96.88	99.54	1.43

^1^ Positive and negative results by a first screening using the commercially available ELISA followed by confirmation by the WOAH IPT assay. ^2^ SE: Sensitivity (number of false positive) and ^3^ SP: Specificity (number of false negative).

**Table 2:** Summary of the ASF-seropositive cases in different phases of pig production from 2019 to 2024.

Year	Num. of Outbreaks in Pig Farms ^1^	Num. of Ab Positive /Num. of Samples	Phases of Pig Production (Antibody Detection)					
			Sow	Gilt	Finishing /Growing	Nursery /Piglet	Boar	Unknown
2019	14	0/238	0/66	0/6	0/81	0/35	0/0	0/50
2020	2	1/50	1/10	0/0	0/20	0/10	0/0	0/10
2021	5	0/145	0/15	0/10	0/80	0/40	0/0	0/0
2022	7	5/308	3/128	0/5	2/145	0/30	0/0	0/0
2023	10	3/690	0/205	0/50	3/265	0/170	0/0	0/0
2024	11	23/681	1/213	3/45	18/364	1/172	0/7	0/0
Total	49	1.43 ^2^ (32/2232)	0.78 (5/637)	2.59 (3/116)	2.41 (23/955)	0.22 (1/457)	0.00 (0/7)	0.00 (0/60)

^1^ Pig farms were confirmed to be infected with ASF through the detection of the ASFV antigen using real-time PCR. ^2^ Positive percentage (%).

**Table 3:** The diagnostic sensitivity, specificity, and kappa coefficient (κ value) of two commercial ELISA kits (bELISA, Ingezim PPA Compac; cELISA, ID.Screen ASF Competition) were evaluated in comparison to the IPT technique, which was used as the gold standard in South Korea.

IPT	bELISA			cELISA		
	Positive	Inconclusive ^1^	Negative	Positive	Inconclusive ^1^	Negative
Positive	25	7	0	29	2	1
Negative	3	2	2195	4	3	641
Total	2232			675		
%SE	100 (25/25)			96.67 (29/30)		
%SP	99.86 (2195/2198)			99.38 (636/640)		
κ value	0.94			0.92		

^1^ Samples with inconclusive ELISA results were considered positive for the purpose of confirmatory testing and were subjected to the IPT.

**Table 4:** Description of the five spatial circular buffers approximated from ASFV-positive wild boar clusters (hotspots) in South Korea from September 2019 to December 2024, including information on both pig farms and wild boars (N = 4256 cases; 49 pig farm outbreaks, 4207 ASF-infected wild boars). The number of farms with positive antibodies within each cluster and the size of the clusters are also provided. The colors correspond to the clusters represented by circles in Figure 2.

Cluster ID	Num. of Wild Boar Points	Num. of Seropositive Pig Farms	Num. of Outbreak Pig Farms	Radius	Color on Map
Cluster 1 (N = 42)	39	1	3	0.287	●
Cluster 2 (N = 32)	28	2	4	0.218	●
Cluster 3 (N = 227)	220	3	7	0.635	●
Cluster 4 (N = 15)	10	2	5	0.165	●
Cluster 5 (N = 613)	608	2	5	0.787	●

## Data Availability

The dataset generated in this study is available from the first author and corresponding author on reasonable request.

## Abbreviations

ASF	African Swine Fever
ASFV	African Swine Fever Virus
IPT	Immunoperoxidase Test
DBSCAN	Density-Based Spatial Clustering of Applications with Noise
WOAH	World Organisation for Animal Health
qPCR	Quantitative Real-Time PCR
PCR	Polymerase Chain Reaction
ELISA	Enzyme-Linked Immunosorbent Assay
bELISA	Ingezim PPA COMPAC
cELISA	ID. Screen ASF Competition
R0	The Basic Reproduction Number
Vero cell	African Green Monkey Kidney Cell
CISA-INIA	Centro de Investigación en Sanidad Animal—Instituto Nacional de Investigación y Tecnología Agraria y Alimentaria
ATCC	American Type Culture Collection
DMEM	Dulbecco’s Modified Eagle’s Medium
FBS	Fetal Bovine Serum
MAFRA	Ministry of Agriculture, Food, and Rural Affairs
APQA	Animal and Plant Quarantine Agency
SOP	Standard Operating Procedure
BSL-3	Biosafety Level 3
x%	Blocking Percentage
S/N%	Competition Percentage
κ value	Hierarchical Kappa-Type Coefficient
